# Influence of Emerging Technologies on the Utilization of Plant Proteins

**DOI:** 10.3389/fnut.2022.809058

**Published:** 2022-02-11

**Authors:** Amanda Gomes Almeida Sá, João Borges Laurindo, Yara Maria Franco Moreno, Bruno Augusto Mattar Carciofi

**Affiliations:** ^1^Department of Chemical and Food Engineering, Federal University of Santa Catarina, Florianópolis, Brazil; ^2^Department of Nutrition, Federal University of Santa Catarina, Florianópolis, Brazil

**Keywords:** plant-based proteins, food processing, eco-friendly technologies, nutritional quality, *in vitro* protein digestibility, food safety

## Abstract

Protein from plant sources is claimed alternatives to animal sources in the human diet. Suitable protein sources need high protein digestibility and amino acid bioavailability. In terms of protein functionality and food applications, they also need high-quality attributes, such as solubility, gelling, water- and oil-holding capacities, emulsifying, and foaming. Thermal processing can improve the nutritional quality of plants with some disadvantages, like reducing the assimilation of micronutrients (vitamins and minerals). Emerging technologies—such as ultrasound, high-pressure, ohmic heating, microwave, pulsed electric field, cold plasma, and enzymatic processes—can overcome those disadvantages. Recent studies demonstrate their enormous potential to improve protein techno-functional properties, protein quality, and decrease protein allergenicity. However, the literature lacks a broader evaluation, including protein digestibility, industrial-scale optimization, and exploring applications to these alternative protein sources.

## Introduction

Proteins are vital macronutrients in human nutrition, supplying the essential amino acids. It performs relevant functional roles in food formulation, processing, storage, and consumption. They also benefit food sensory and quality attributes, depending on their functional properties, such as solubility, gelling, water- and oil-holding capacities, emulsifying, and foaming (1, 2).

The main current challenges regarding protein food sources are supply and distribution guarantees. Regarding animal protein production and consumption, there are concerns about adverse impacts on human health, natural resource depletion, climate change, and animal welfare (3). These concerns lead to a constant growing adoption of vegetarian and vegan diets. Food security awareness for the increasing world population (about 10 billion by 2050) drives the demand for sustainable protein sources (4). The most prominent new protein sources are non-conventional plants (including agro-industrial by-products), fungi, algae, and insects (5, 6). Although food-grade insects are excellent protein sources, they are not widely accepted by consumers, mainly due to cultural aspects. Fungi and algae are limited in terms of supply. Thus, plant-based proteins keep drowning the most attention among others.

In general, thermal processing improves plant protein nutritional quality. However, some disadvantages are high time- and energy-consuming procedures, large water expenditure, and losses of desirable compounds in the final product (7). The main chemical changes produced by heating are degrading heat-labile micronutrients, reducing vitamins and minerals assimilation, and when the Maillard reaction occurs, generating toxic compounds and reducing the essential amino acids bioavailability (8). Elevated processing temperatures may also induce crosslinking, protein-protein interactions, and amino acid racemization (9).

**Figure 1:**
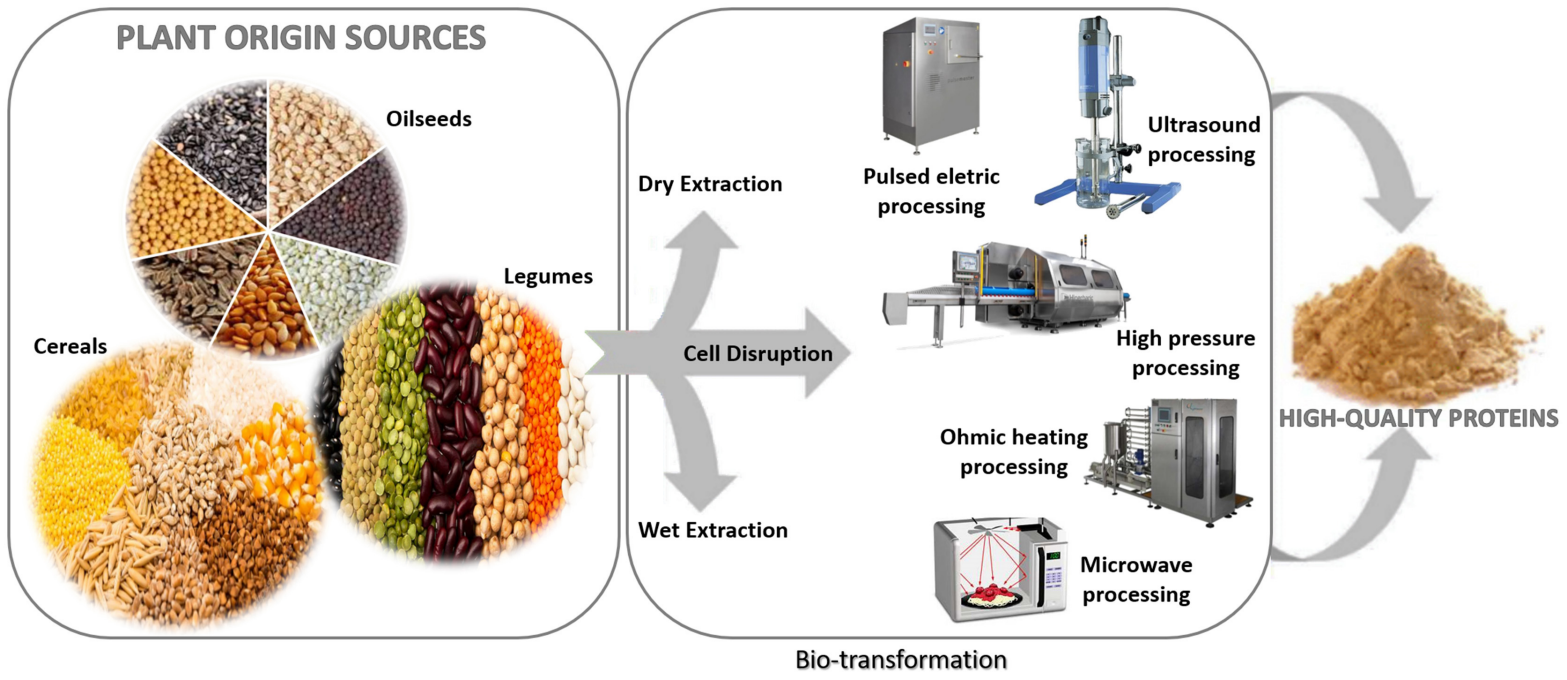
Emerging technologies for protein valorization of plant origin sources. Adapted from Pojić et al. (11).

Alternatively, some emerging food processing technologies have been investigated for the best protein employment, such as ultrasound, microwave, supercritical fluids, pulsed electric field, high-pressure, ohmic heating, cold plasma, and enzymatic processes (10, 11). Figure 1 illustrates the use of these techniques for valorizing plant-based proteins (11), which may contribute to environmental preservation by reducing wastewater production, organic solvents utilization, and processing time (12). Under mild temperatures, a balance can be reached between the processing feasibility with reduced environmental impact and the increased nutritional aspects and techno-functionalities of the proteins.

Plants have been studied and used worldwide as protein sources, including legumes, cereals, pseudocereals, and seeds (6). However, plant-based proteins are negatively associated with a diminished nutritional quality due to minor components that impair protein bioavailability to the human body. These antinutritional factors are protease (trypsin) inhibitors, polyphenols (tannins), phytates, fibers, haemagglutinins, and non-starch polysaccharides (8). Thus, a proper plant protein processing selection may affect the digestibility and nutritional value by inactivating or eliminating these compounds, modifying the profile of bioactive peptides, and changing the protein structure (13). Besides, some plant proteins present food allergenicity, including nuts, wheat, and soybean (14). Those emerging technologies are also being investigated to create hypoallergenic products (15) due to changing the protein conformation and the IgE epitopes of the allergens, making them less available to antibody receptors, which declines the protein allergenicity, as well as increase the protein digestibility (16).

Therefore, this critical review aims to discuss the advances and perspectives of the emerging processing methods—non-thermal or performed at mild temperatures/short times—to improve plant protein quality and properties. It is focused on the nutritional aspects, protein digestibility, protein allergenicity, and techno-functional properties.

## Techno-Functional Properties of Plant Proteins

Protein functionality is critical in determining the applicability of plant proteins flours, concentrates, and isolates (17, 18). The bio-functionality is related to protein physiological and nutritional properties (e.g., antioxidant and antibacterial activity) (11). On the other hand, techno-functionality is associated with the impact on the physicochemical characteristics of food products, affecting the texture, appearance, stability, emulsifying, solubility, foaming, gelling, water- and oil-holding capacities, cohesion-adhesion, elasticity, and viscosity (19).

Table 1 presents some protein techno-functional properties and their relationship with food sensory and physicochemical characteristics.

**Table 1:** Functional properties and their relationships with physicochemical and sensory properties of proteins (15, 49, 106, 107).

**Functional property**	**Definition**	**Physicochemical property and mode of action**	**Sensory property**	**Examples of plant proteins**	**Products and food systems**
Solubility	Interaction of protein surface hydrophilic groups with water	Hydrophilicity, H-bonding and surface ionization, protein solvation, pH-dependent	Flavor, taste, mouthfeel, turbidity	Soybean, almond and rice proteins	Beverages
Foaming	Formation films to entrap air and foam stabilization	Hydrophilicity, Hydrophobicity, film formation in the air/water interface	Mouthfeel, smoothness	Seeds protein	Desserts, ice cream, cakes, mousses
Emulsifying	Formation and stabilization of emulsions	Hydrophilicity, Hydrophobicity, film formation in oil/water interface	Mouthfeel, flavor, smoothness	Seeds protein	Meat analogs, soups, sauces, desserts, cakes, ice cream, salad dressings
Gelling	Capacity to form gels	Thermal aggregation, water entrapment, and immobilization, protein matrix formation	Mouthfeel, texture, smoothness	Seeds protein	Deserts, meat analogs, and bakery products
Oil-holding capacity	Fat entrapment	Hydrophobicity	Flavor, odor, smoothness	Seeds protein	Beverages, sauces, meat analogs, bakery products
Water-holding capacity	Water entrapment	Ionic hydration, H-bonding,	Texture, consistency	Soybean and cereal proteins	Meat analogs, cakes, bakery products
Viscosity	Thickening	H-bonding, hydrodynamic shape, and size, water-binding,	Taste, consistency, mouthfeel	Soybean	Soups, salad dressings, sauces, deserts
Elasticity	Stretchiness	Hydrophobicity, disulfide crosslinking deformable gels	Texture, crispiness, chewiness	Gluten protein	Meat analogs, extruded and bakery products
Cohesion and adhesion	Protein acts as an adhesive material	H-bonding, Ionic-bonding	Chewiness, stickiness	Seeds protein	Meat analogs, pasta, extruded snacks, and bakery products

Food macromolecules (e.g., polysaccharides, lipids, and proteins) are inherently functional by their molecular structure and ability to interact and form complexes. Protein molecular structures have important roles in determining food functionality and can be used as targets to alter protein functionality (20). Intrinsic and environmental factors determine the functional properties, stability, and shelf-life of foods containing functional proteins. The main intrinsic factors are the protein structure, conformation, amino acid composition, surface functional groups, net and surface electric charge, and hydrophobicity/hydrophilicity. Extrinsic factors are the medium pH, salts and solvents, ionic strength, temperature, pressure, and shear stress (21, 22). Protein extraction and processing may change those functional properties. Thus, it is also essential to study the parameters setup impact on a diversity of functional and physicochemical properties of food products.

Most proteins are functional due to their globular component properties, especially solubility, which is attributed to the amphiphilicity of these molecules. Proteins have both inwardly bounded apolar (hydrophobic) amino acids and outwardly exposed polar (hydrophilic) side-chain amino acid residues. This arrangement allows dipole-dipole interactions with solvents by twisting and unfolding the amino acid side chains, placing the polar groups at the protein's surface. It leads to networks that can form gels and develop films, hold water, absorb fat, foam, emulsify, and dissolve under various pH conditions (20). Also, the relative amount of α-helices, random coils, and the α-helix/β-sheet ratio in protein secondary structures of soybean and corn meals were positively correlated with protein solubility, while the percentage of β-sheet structures was negatively correlated with this same ability (23).

Some studies assessed the techno-functional properties of plant proteins, such as soybean, chickpea, kidney bean (24), mung bean, pea (22, 25), cowpea, lentils (26), amaranth, quinoa (16), cashew nut (27), sorghum (28), avocado (29), and mustard (30). Few studies investigated these properties in edible oil processing by-products, such as rapeseed meal protein solubility (31, 32). The utilization of plant proteins is limited due to their extremely low solubility at neutral pH, except for the soybean, pea, canola (9), and cowpea (33).

Other studies also evaluated some plant proteins' **foaming** capacity and stability, like soybean, pea, chickpea, lupin, and rapeseed (34, 35). These sources have excellent foaming properties, comparable to egg protein, mostly due to high solubility, high surface hydrophobicity, low molecular weight, and net charge (36).

Some plant proteins have highlighted **emulsifying properties**, like the bell pepper, which formed stabilized emulsions with small oil droplet sizes (37); peas (34); chickpeas, with high emulsion activity index (EAI) at pH 10 (35); soybean, with a high emulsifying capability and emulsion stabilization against creaming during storage (38); and rapeseed, with higher emulsifying stabilities than soybean products (39).

Furthermore, few studies evaluated the **water- and oil-holding capacities** (WHC and OHC) of plant proteins. Bell peppers are suggested to food products requiring high WHC (37), while the OHC of peanut protein isolates was remarkably higher than commercial soybean protein isolates (40).

Although few studies evaluated the **gelling properties** of plant proteins, there are results about rapeseed products (flours, concentrates, and isolates) reporting poor gelation properties (41). However, soybean protein isolates have been used as gelling agents in several semi-solid food products, mainly for meat analogs (42).

Therefore, in terms of techno-functionality, there is little research reporting the solubility, emulsifying, foaming, water- and oil-holding capacities, and gelling properties for plant proteins. Potentially, plant-based proteins may be used by the food industry in formulations for protein supplements, meat analogs, beverages, snacks, desserts, bakery, whipped creams, soups, sauces, and salad dressings (22). From here, one can consider that exploring plant-based proteins aiming to develop technological alternatives for food formulation is an open field, including evaluating the required processing technologies for extraction and modulating thetechno-functionalities.

## Emerging Technologies for Protein Valorization, Recovery, and Improvement of Protein Quality

The employability of plant proteins is related to the availability of their use, in addition to their intrinsic properties. The extraction yield from a food source is related to protein structure (primary to quaternary). Withal, the complexity of the food matrix influences the extraction process: proteins are generally bonded to other macromolecules (e.g., carbohydrates); the presence of salts and different pH values change proteins' charge, ionic strength, conformation, and solubility; previous matrix processing (e.g., defatting or heating) and the presence of water/solvents alters the matrix structure; and the fractionation of the plant sources (43–46). Also, the structural, functional, and sensory properties of extracted proteins are influenced by the conditions under which plant proteins are processed, e.g., temperature, time, pH, and ionic strength (44).

**Figure 2:**
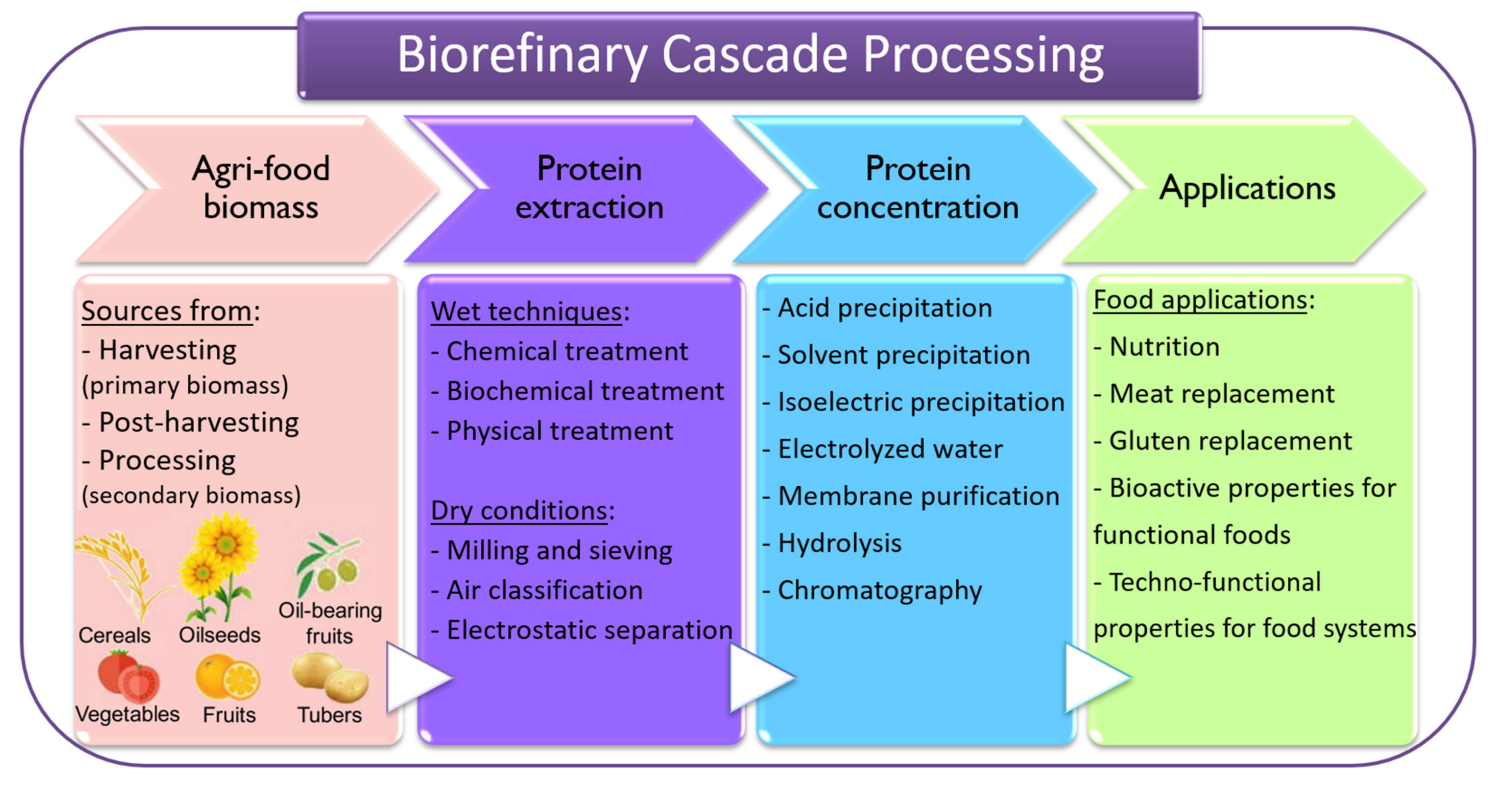
Flow diagram representing the extraction methods most used for recovering proteins from agri-food. Adapted from Contreras et al. (9).

Protein extraction can be categorized into wet and dry methods (11). Subsequently to the extraction step, many technologies can be employed to purify or concentrate the protein of interest, aiming to obtain a food ingredient for different purposes. Figure 2 presents a diagram with the methods most used for recovering proteins from agri-food materials, which could be applied in a biorefinery concept.

The main consequence of many extraction methods is protein denaturation, which alters the proteins' secondary, tertiary, and quaternary structures due to changes in temperature, pH, and organic solvents and salts presence (47). In general, denaturation makes the protein more susceptible to digestive enzymes, improving protein digestibility (21).

Alkaline extraction (pH 8–11), followed by isoelectric precipitation (pH 4–5) of solubilized proteins, is the most usual technique employed for the extraction of plant proteins to make enriched flours (up to 65% of protein), protein concentrates (65–90%), and protein isolates (more than 90%) (48). Several studies used the alkaline technique to produce protein isolates of plants, such as seeds, cereals, and legumes (9). However, the enormous water, energy, and chemicals requirement is a significant drawback of alkaline extraction (19). Besides, high protein purity and yield are not guaranteed, which are affected by the processing conditions (e.g., equipment configuration, extraction time, temperature, pH, ionic strength, net charge, presence of salts, protein content, and protein solubility) (26, 49). Also, extreme extraction conditions (e.g., highly acid or alkaline medium and high temperature) may reduce protein nutritive value by changing the amino acid profile and degrading bioactive compounds (9, 11, 50). Furthermore, acid precipitation and neutralization tend to reduce protein solubility and negatively impact other techno-functional properties, such as gelling and foaming (51). Therefore, the alternative technique using membranes (e.g., ultrafiltration) is a less-energy consuming alternative to concentrate proteins instead of isoelectric precipitation, reaching the manufacture of protein ingredients (21, 26) and resulting in improved protein recovery yield, preserved techno-functional protein properties, and higher purity.

The existing drawbacks of conventional extraction methods (e.g., high temperature, energy consumption, wastewater, and organic solvents utilization) (19) may be overcome by emerging technologies using mild conditions. The most prominent alternatives are ultrasound, high-pressure, microwave, pulsed electric field, ohmic heating, and enzymatic processes. They potentially increase the protein extraction yield while reducing chemicals and water consumption (9–11, 50, 52, 53). However, a key question is whether these novel technologies can extract proteins from agri-food sources efficiently and cost-effectively. Data about the required energy and costs are scarce, and these approaches were mainly performed at a laboratory scale or are still in the early stage of their industrial applications; therefore, the large-scale feasibility still needs further studies.

Although these emerging processes were evaluated about protein valorization and recovery, studies concerning protein quality improvement (e.g., protein digestibility and inactivation of the so-called “antinutritional factors”) are rare for plant protein sources. Few evaluations have been made for the inactivating trypsin inhibitors in plant proteins in soybeans, chickpeas, and beans (54). However, as presented in the following, reasonable indications are that those methods are alternatives for plant-based protein processing.

### High-Pressure Processing

High-pressure processing (HPP)—also known as high hydrostatic pressure (HHP) or high isostatic pressure (HIP)—is a non-thermal technology using hydrostatic pressures up to 1,000 MPa into a product in controlled temperature and time conditions (55). HPP affects the structure of the non-covalent bonds, increases the surface hydrophobicity, and causes protein denaturation, aggregation, or gelation (56). The protein secondary and tertiary structures significantly change at pressures higher than 200 MPa due to the consequent denaturation and aggregation of plant proteins with increased pressure, which changes the conformation and coagulation of their native structures because of the disruption of interactive forces, mainly hydrophobic and electrostatic bonds (57). HPP also improves protein functionality and digestibility of cereals and legumes (58), inactivating the antinutritional factors on a laboratory scale (59).

High-pressure homogenization (HPH)—also called dynamic high-pressure (DHP) or ultra-high pressure homogenization (UHPH)—imposes high-pressure conditions by pumping liquid food through a tiny gap in a valve, increasing velocity and causing high shear stresses. Consequently, it causes changes in food rheological properties. HPH utilizes the combined effectiveness of high-frequency vibration, high-velocity impact, quick pressure drop, cavitation, and intense shear stress in a short time, which causes a significant effect on proteins conformation (60). HPH was also recently applied to food products aiming at microbial inactivation and changes in the protein techno-functional properties (55).

Typical HPH pressures are moderate and usually up to 100 MPa (61), while HPP can reach ten times more. In addition to the range of applied pressures, another difference between HPP and HPH is the molecules' movements during treatment (lower in HPP and increased in HPH), leading to different protein structures after the treatments, bringing many interaction possibilities between polypeptides and protein aggregation. Also, HPP is governed by the ordering principle (33), whereas during HPH, high shear forces perturb protein structures (62).

Table 2 shows examples of high-pressure technology applied to plant proteins. The use of elevated pressure favors extracting protein from plants, decreasing solvent consumption, increasing extraction yields, and shortening the extraction time. High-pressure can modify protein techno-functional properties and reduce protein allergenicity (63, 64). HPP has also been used to extract proteins from some agri-food residues (e.g., wheat bran, grape pomace, and corn stover) (9), reduce food allergenicity, and inactivate some compounds detrimental to protein digestion.

**Table 2:** Examples of emerging technologies application on plant proteins.

**Process**	**Objective of the study**	**Processing conditions**	**Results**	**Protein yield**	**References**
**HPP**					
Soybean	Protein allergenicity	350 MPa 20°C 16 min	Reduced allergenicity by 46.6%	^*^	(64)
Soybean protein isolate	Reduction of antinutritional factors	200–700 MPa 20°C 20 min	Efficient to eliminate the phytates, however, not effective to reduce trypsin inhibitor	^*^	(108)
Soybean protein isolate	*In vitro* protein digestibility	400–600 MPa 20°C 20 min	Increased IVPD 68%	^*^	(109)
Soybean protein isolate	Functional properties	100–300 MPa	Foaming increased and viscosity decreased	^*^	(110)
Soybean slurry (by-product)	Protein extraction	50–125 MPa	Good results of extraction yield at 100 MPa	82%	(65)
Kidney bean protein isolate	Functional properties	300–600 MPa 15 min	Production of isolate with higher functionality	23.5%	(10)
Peanut protein isolate	Functional properties	50–200 MPa 5 min	Improved water- and oil-holding capacities, but not improved gelling property	^*^	(40)
Sweet potato protein	*In vitro* protein digestibility	200–600 MPa 20 min	Increased IVPD from 53.8 to 59.1% in 30 min	^*^	(111)
Sweet potato protein	Functional properties	250–550 MPa pH 3–9	400 MPa was a good choice for preparing novelty food products with structural modification	^*^	(112)
Sweet potato protein	Gellation behavior	400 MPa 25°C 30 min	Textural properties of gels were improved by sulfur-containing amino acids, especially by cysteine	^*^	(56)
Macuna bean protein isolate	Protein extraction and color evaluation	200–600 MPa 20 min pH 6.37	Inactivation of polyphenol oxidase and improvement of the color of protein isolate	8–34%	(113)
Pea protein isolate	Functional properties	200–600 MPa 23°C 5 min	Improvement of emulsion and foaming capacities	^*^	(114)
Fababean	Functional properties	103–207 MPa 32–45°C 6 cycles	Improvement in solubility and foaming capacity and decreased emulsifying capacity	^*^	(115)
Lentil protein isolate	Functional properties	34–103 MPa 4 cycles	Decreased surface hydrophobicity and increased zeta potential	^*^	(116)
Hazelnut	Functional properties	25–150 MPa 25°C	Improvement of solubility, foaming, emulsifying capacity, and emulsifying stability	^*^	(117)
Mung bean, chickpea, pea, lentil, and faba bean yogurts	Rheological analyses	600 MPa 5 min	Viscosity and viscoelastic properties of plant protein gels was comparable to commercial dairy yogurts	^*^	(118)
Potato protein isolate	Gelation properties	300–500 MPa	High pressures can allow the formation of gels from potato protein isolate as a novel plant-based protein source	^*^	(119)
Cowpea	Gelation properties	400–600 MPa 5 min	HHP-induced gels were less hard and adhesive than heat-induced ones	^*^	(33)
**US**					
Soybean	Inactivation of trypsin inhibitor	20 kHz 20 min	Inactivation of trypsin inhibitor by 55%	^*^	(120)
Soybean protein isolate	Emulsifying property	200–600 W	Improved emulsifying capability	^*^	(38)
Soybean protein isolate	Gelation properties	20 kHz 150–450 W	Under 300 W, the gel hardness reached a maximum of 998.9 g, with water binding capacity of 87%	^*^	(121)
Soybean okara (by-product)	Protein extraction	20 kHz 65 W 15 min	US improved the extraction of up to 10%	70%	(66)
Soybean milk	*In vitro* protein digestibility and inactivation of trypsin inhibitor	25 kHz 400 W 1–16 min	US significantly reduced trypsin inhibitor activity up to 52% and improved the digestibility of proteins in soymilk	^*^	(122)
Millet protein concentrate	Functional properties	20–100 W 18.4–73.9 W/cm^2^ 5–20 min	Improvement of solubility and emulsifying capacity	^*^	(71)
Pea protein concentrate	Functional properties	412.5–712.5 W 336–582 s	Emulsifying properties were greatly improved	^*^	(18)
Pea protein isolate	Foaming property	20 kHz Amplitude of 30–90% 30 min	Foaming ability increased from 145.6 to 200% and foaming stability increased from 58 to 73.3%	^*^	(25)
Soybean and rice protein isolates and pea protein	Functional properties	20 kHz 562.5–712.5 W 120–600 s	Functional properties are improved as the dispersibility of protein materials increases (712.5 W, 600 s)	^*^	(123)
Potato protein	*In vitro* protein digestibility and functional properties	20–60 kHz 2–30 min 40°C	Solubility and digestibility of potato protein was significantly improved by online ultrasound-assisted pH shifting treatment	^*^	(124)
Barley protein isolate	Functional properties	20 kHz 100% amplitude	Improved protein solubility and colloidal stability especially at alkaline pH	^*^	(125)
Sunflower protein isolate	Functional properties	20–40 kHz 5–30 min	Improved solubility, emulsifying, foaming and oil-holding capacity and decreased water-holding capacity	^*^	(126)
Olive kernel	Protein and phenolic compounds extraction	400 W 24 kHz 100% amplitude	Potential use for protein extraction	25%	(67)
Tamarind seed protein isolate	Functional properties	100–200 W 15–30 min	Solubility, emulsifying, foaming capacity, water- and oil-holding capacity was the highest when both time and intensity of treatment were high	^*^	(127)
Bell pepper seed protein isolate	Protein extraction and functional properties	350 W	High oil-holding capacity, low solubility, and low foaming property	6%	(37)
Pea	Functional properties	68 W/100 mL 20 kHz	Both pH-shifting at pH 12 and power ultrasound treatments were effective in modifying the properties of pea	^*^	(128)
**PEF**					
Grape juice	Impact on the protein structure	35 kV/cm 4 μs pulses at 1,000 Hz	No evidence that PEF affects the primary structure of proteins and amino acid content	^*^	(129)
Rapeseed stems and leaves	Protein and polyphenols extraction	0.2–20 kV/cm	Enhanced protein yield	80%	(130)
Alfafa leaves	Protein extraction	^*^	Increase of protein extracted by PEF	57%	(131)
Olive kernel	Protein and phenolic compounds extraction	Pulse voltage of 40 kV	Increased the total phenolic content and proteins of the recovered extracts	25%	(67)
Pea, rice, and gluten protein concentrates	Functional properties	60,000 pulses 1.65 kV/cm	PEF was able to modify protein structure by inducing unfolding, intramolecular rearrangement, and formation of aggregates. These effects were strongly dependent on protein nature and pH	^*^	(68)
Blackberries	Protein and phenolic compounds extraction	40 kV−10 kA 0.5 Hz 13.3 kV/cm	The maximum anthocyanin yield was found after applying PEF treatment	38 mg/100 g	(69)
**MH**					
Beans	Inactivation of trypsin inhibitor	2,450 MHz 5–20 min	Effective for inactivation of trypsin inhibitor (97–100%) of different varieties of beans	^*^	(132)
Soybean	Inactivation of trypsin inhibitor	2,450 MHz 500 W 2–4 min	Trypsin inhibitor was completely inactivated	^*^	(45)
Soybean milk	*In vitro* protein digestibility and inactivation of trypsin inhibitor	2.45 GHz 70–100 °C 2–8 min	Increased digestibility by 7% Trypsin inhibitor activity reduced to 1%	^*^	(54)
Soybean milk	*In vitro* protein digestibility and inactivation of trypsin inhibitor	2,450 MHz 70–100°C 2–10 min	Digestibility of soymilk significantly increased up to 93% after 10 min microwave processing at 85°C	^*^	(122)
Soybean milk	Protein extraction, digestibility and functional properties	540–810 W 70–90°C 140–180 rpm	The optimal microwave-assisted extraction conditions were 675 W, 80°C and 160 rpm	24%	(133)
Rapeseed meal	*In vitro* protein digestibility	800 W 2–6 min	Microwave for 2 and 4 min increased IVPD and for 6 min decreased IVPD	^*^	(100, 134)
Peanut peptides	Degree of hydrolysis	9.5 min 600 W 50°C	DH reach 26.1%	^*^	(135)
Chickpea	Comparison of process time with conventional methods	400–600 W 14–56 s	Reduction of cooking times from microwave	^*^	(76)
Chickpea	*In vitro* protein digestibility and inactivation of trypsin inhibitor	2,450 MHz 15 min	IVPD were improved, and trypsin inhibitor activity was significantly decreased	^*^	(136)
Coffee silverskin protein (by-product)	Protein extraction	434.7 W 10–20 min	Microwave-assisted extraction have potential to be a rapid and effective tool for protein extraction from coffee silverskin	43.53%	(46)
**CAPP**					
Pea protein isolate	Functional properties	Air DBD 8.8 kVPP 3 kHz 10 min	Improvement of protein solubility, water- and oil-holding capacities	^*^	(88)
Peanut protein isolate	Functional properties	DBD 35 kV 1–4 min	Improvement of emulsion stability, solubility, and water-holding capacity	^*^	(137)
Wheat flour	Functional properties	Air 20 V 9 kHz 120 s	Increase in the dough strength	^*^	(138)
Soybean protein isolate	Functional properties and allergenicity	DBD 40–60 kV 80–100 kHz 1–10 min	CAPP induced reactive oxygen species resulting in modifications in the secondary and ternary structures. Functional properties such as emulsifying and foaming properties (60 to 194%) were influenced. The IgE-binding level was decreased by up to 75% (120 Hz, 5 min)	^*^	(83)
Rice flour	Amino acid composition	DBD 60–70 kV 5–10 min	Higher content of amino acids for samples treated with cold plasma (glutamic acid, asparagine, serine, histidine, threonine, tryptophan, isoleucine, phenylalanine, and proline)	^*^	(139)
Wheat grain and flour	Functional properties	DBD 80 kV 5–30 min	Plasma treatment increased the flour hydration, pasting and viscosity properties of wheat flour	^*^	(86)
**EAEP**					
Soybean	Protein extraction	Protease M^®^ pH 4.5 50–100°C 10–120 min	Good results of protein yield	59.3%	(140)
Peanut	Protein extraction	Alcalase^®^ 1.5% 60°C pH 9.5, 5 h	Good results of protein yield	71.4%	(141)
Sesame bran	Protein extraction	Viscozyme L.^®^ Alcalase^®^ 25–55°C 10–120 min	Good results of protein yield	88.8%	(50)
Rice bran	Protein extraction	Alcalase^®^ 50°C	Good results of protein yield	44.8%	(142)
Oat bran	Protein extraction and functional properties	Amylogluciosidade 55°C pH 11.5, 60 min	Good results of protein yield	82%	(143)
*Moringa oleifera* seed	Protein extraction	Protex 7L^®^ 45°C 15 min	Good results of protein yield	75.4%	(144)
Rapeseed meal	Protein extraction	Viscozyme^®^ Alcalase^®^ 80 min	Good results of protein yield	82.1%	(145)
Almond cake	Protein extraction and protein digestibility	FoodPro Alkaline Protease^®^ 50°C pH 9.0 120 rpm 1 h	64% of protein digestibility (almond skim fraction)	^*^	(146)
**EH**					
Palm kernel cake	Improve nutrient utilization	Mannanase 1–20% 2–12 h	Mannanase improved nutrient release of reducing sugar, total sugar and proteins	^*^	(147)
Chickpea protein isolate	Functional properties	Alcalase^®^ pH 8.0 50°C 210 min	Improvement of protein recovery, solubility, and emulsifying properties	^*^	(148)
Peanut protein isolate	Functional properties	Papain 130°C	Enhanced DH and increased protein solubility	^*^	(97)
Beans	*In vitro* protein digestibility	Proteases 28°C 150 rpm 5 h	Enzyme treatment improved the IVPD of the four bean varieties	^*^	(149)
Lupin protein isolates	Functional properties	Alcalase 2.4L^®^, Papain^®^, Corolase 7089^®^, and Neutrase 0.8L^®^	The enzymatic hydrolysis increased their techno-functional properties (protein solubility, foam activity, and emulsifying capacity) independently of the enzyme preparation	^*^	(99)
**DSI**					
Pea and rice protein isolate	Functional properties	107°C pH 9–11	Enhanced solubility, emulsifying, foaming, and gelling for protein treated by DSI	^*^	(101)
**RW**					
Chickpea protein isolate	Functional properties	90°C 20 min	RW samples had a better water-holding capacity and emulsifying stability compared to freeze-drying samples	^*^	(35)
**GI**					
Sunflower meal	*In vitro* protein digestibility	10–20 kGy	Improved the IVPD (85.5%)	^*^	(100)
Rapeseed	Phytic acid concentration	15–45 kGy	100% inactivation of phytic acid	^*^	(100)

* *Data not found in the respective study*.

*CAPP, Cold-atmospheric pressure plasma; DBD, Dielectric barrier discharge; DH, Degree of hydrolysis; DSI, Direct steam injection; EAEP, Enzyme-assisted extraction processing; EH, Enzymatic hydrolysis; GI, Gamma irradiation; HPP, High pressure processing; IVPD, in vitro protein digestibility; MH, Microwave heating; PEF, Pulsed electric field; RW, Refractance-window; US, Ultrasound*.

### Ultrasound

Ultrasound (US) is a non-thermal technology using high-intensity and low-frequency sound waves, ranging from 20 to 100 kHz (11). The basic principle of ultrasound technology is the cavitation phenomenon, where air bubbles are formed within the liquid phase, their volume increase, and finally explodes (19). US accelerates the mass transfer of compounds, provides high shear forces in the extractive agent (50), and improves solubility due to cellular structure's high stress and deformation (19). The mass transfer is also facilitated because microchannels may occur when bubbles collapse. The increased temperature, turbulence, and mixing effects by cavitation in the US also increase extraction efficiency (19). Consequently, the US can modify proteins by affecting H-bonds, increasing protein recovery, and reducing extraction time and protein aggregates (9). Also, improved protein functionality can occur. The cavitation bubbles on the protein surface result in micro-jetting and particle breakdown, improve solvent permeation into the food matrix and change the protein allergen conformation and reactivity (9, 11).

As a novel technology, the US appealed to environmental sustainability. High-intensity ultrasound is a quick and cost-effective technology used to modify globular proteins' structural and functional properties (25). The US was used to recover valuable proteins from food industry by-products, e.g., soybean okara (65, 66) and olive kernel (67). Table 2 summarizes the US technology application to plant proteins.

### Pulsed Electric Field

**Figure 3:**
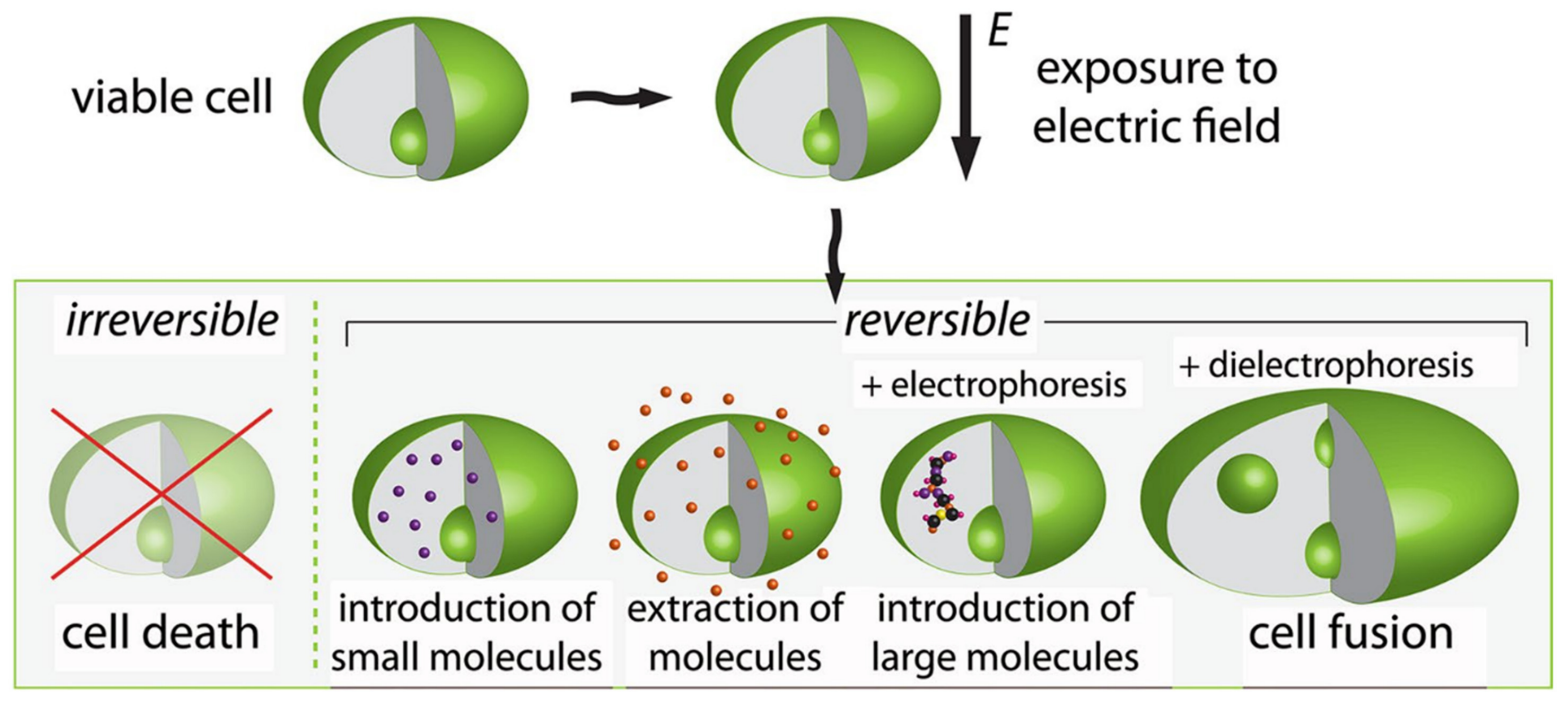
Schematic representation of the effect on cells exposed to pulsed electric fields. Adapted from Golberg et al. (12).

Pulsed electric field (PEF) treatment consists of electric pulses of short duration (10^−4^ to 10^−2^ s) and relatively high amplitude (0.1–80 kV/cm). It induces a critical electrical potential across cell membranes, enabling an easier extraction of proteins (9, 11). PEF is a promising non-thermal food processing method that is efficient in cell disintegration (12) and microbial inactivation. PEF also induces changes in the protein hydrophobicity and structure (secondary and tertiary) and dissociates the non-covalent bondings, improving the protein functionality (68). PEF technology may cause lethal damage to cells or induce sub-lethal stress by transient permeabilization of cell membranes and electrophoretic movement of charged species between cellular compartments. Therefore, PEF application can facilitate the selective recovery of valuable compounds without deteriorating the treated matrix, favoring the subsequent separation and purification stages (69). Exposure of viable cells to PEF increases cell membrane permeability due to electroporation. This phenomenon can be used in biorefineries to extract and introduce molecules into the cells. The PEF application on plant cell tissues can positively change the membrane transport properties, facilitating the extraction of targeted molecules (e.g., proteins). Figure 3 schematically represents the impacts of PEF on any viable cells, where the possible outcomes depend on the PEF setup protocols (amplitude, number, shape, and pulses duration) and additional techniques (e.g., electrophoresis) (12).

The PEF advantages are increased mass transfer, improved extraction yield, decreased processing time, reduced compounds degradation (e.g., flavors and proteins), and reduced energy costs (68). The application of PEF is a potential alternative to recover high-added value compounds from food matrixes and residues, which reduces waste disposal and extends limited resources. However, these studies were performed at the laboratory scale, and further investigations are needed to address the large-scale feasibility and energy costs.

Recent studies used PEF on the recovery of bioactive compounds from plants (e.g., betalain from red beet, beta-carotene from carrot, sucrose from sugar beetroot, inulin from chicory, and phenolics from grapes), agri-food residues, such as pomace, peels, kernels, and oilseed meals (e.g., carotenoids, chlorophylls, sterols, and polyphenols), and marine microalgae without killing the cells (12, 49, 70). However, the literature using PEF to improve protein digestibility and reduce antinutritional factors of plant proteins is scarce, and further research on this topic may be worthy. Table 2 presents applications of PEF to plant proteins.

### Ohmic Heating

Ohmic heating (OH) is an advanced thermal processing method that applies electrical current to generate heat inside a food material by the well-known Joule effect (71). OH emerged as an alternative method to food thermal pasteurization and sterilization (11) and can promote higher yields and lower processing time than the conventional methods, bringing significant nutrient retention and preservation of the food quality (60). The existence of electrolytic components, such as salt and acids, allows the electric current to pass through food materials, which is the basis of the OH technique, generating heat internally (72). Besides heating, OH causes electroporation of cell membranes, increases the electrical conductivity and permeability, and positively influences the extraction rates of different biomolecules (11).

It was shown that OH inactivates trypsin inhibitors due to the electrochemical effects, depending on the electric voltage used. Studies using OH (50 Hz, 220 V, 3 min) showed more efficient inactivation of the trypsin inhibitors than the electric stove over the same processing time (59). The heat produced by the OH depends on the food material's electrical field strength and electrical conductivity. Low electrical conductivity heats slower than higher ones if the same electrical field strength is used. Food materials with 0.01–10 S/m of electrical conductivities are considered proper for the OH technique (73). OH was a pretreatment to soybean oil recovery, in which 600 V for 10 min promoted a 73% yield at 90°C (74). However, no studies using OH to assist protein extraction or improve plant proteins' functional properties and digestibility were found. Thus, there are opportunities for new applications for this emerging technology.

### Microwave

Microwave heating (MH) uses non-ionizing electromagnetic waves from 300 MHz to 300 GHz (75). Microwaves cause heating due to the interaction of the alternating electromagnetic field with the food chemical constituents. MH of food materials occurs due to dipole and ionic mechanisms, and the dielectric properties and penetration depth are the most important characteristics affecting the process (76).

Microwave is mostly applied to foods for shorting cooking time, with less energy consumption. However, this technique can also influence the extraction of proteins (46) and other nutrients in food matrix (e.g., polyphenols and polysaccharides) (77). Moreover, the advanced MH use may improve the nutritional food quality by combining operating conditions that preserve more nutrients (e.g., vitamins and heat-labile amino acids like lysine, tryptophan, and sulfur amino acids) and sensory aspects (78–80). MH affects the conformational properties of food proteins (secondary structure) and accelerates their denaturation without changing their primary structure (60). The microwave processing disrupts H-bonds, increases the food matrix porosity, and allows dissolved ions migration. These food changes facilitate the extraction and the improvement of the protein functional properties (11). MH is also useful for inactivating the antinutritional factors usually present in plant proteins (59) and improves protein digestibility (8). Table 2 presents MH applications to plant proteins.

Literature shows some studies that use microwave heating to reduce the cooking time of plants, reduce the concentration of antinutritional factors, and enhance protein digestibility (8). However, there is scarce evidence of efficiently using this technology to extract proteins from plant sources and agro-industrial waste.

### Cold Plasma

Plasma technology (or gas discharge plasma) involves producing and using ionized gas molecules to treat a material for superficial effects, e.g., polymers functionalization and food decontamination. The gas ionization produces reactive species, e.g., ions, electrons, excited atoms, ultraviolet (UV) photons, and free radicals (81–83), that can cause different changes on the food surfaces (solids food) and food bulk. Plasma can be generated at different temperatures and classified into thermal and non-thermal (also called cold) plasma (81, 84). Depending on the pressure condition, plasma can also be classified as low-, high-, or atmospheric-pressure plasma (85).

Cold atmospheric plasma processing (CAPP) is an emerging, sustainable, and environmentally friendly technology that has received significant attention in the food industry for the recent decade (82, 83, 86). CAPP can be generated by corona discharge, atmospheric-pressure plasma jet (APPJ), dielectric barrier discharge (DBD), microwave discharge (85, 87), and radiofrequency (RF) (88). It produces reactive oxygen species, including singlet oxygen and ozone, and exciting molecular nitrogen (81, 89). CAPP has an inactivation effect on microorganisms, such as food pathogens, spores, and viruses (84, 85, 89). Also, it is valid for surface modification (90, 91), inactivation of deteriorating enzymes (82), food packaging modification (85), and reduction of food allergenicity (83). According to processing conditions, CAPP influences food proteins' conformation and functional properties, e.g., plasma source, design, treatment power, pressure, reactive gas type, exposed time, and sample nature (84, 85).

Reactions initiated by reactive oxygen species (ROS) with the synergistic effect of reactive nitrogen species (RNS) are the leading cause of protein structure modification, triggering the cleavage of proteins into peptides (84). Also, CAPP can damage proteins, amino acids, nucleic acids, and lipids (81, 84, 89). The plasma protein denaturation mechanism is associated with reactive species interaction with amino acids and secondary structure by losing α-helix and β-sheet (85). Besides, it was observed that sulfur and aromatic amino acid content decreased after plasma exposure (84, 92).

There are few studies regarding CAPP effects on protein modification and the techno-functional properties of plant proteins and by-products. Table 2 brings some applications of CAPP technology to plant proteins present in the current literature. There is no published report on the extraction and protein quality enhancement of plant proteins in terms of protein digestibility by plasma technology. Also, no studies about the protein recovery of plant proteins from by-products using CAPP were found in the literature.

### Enzymatic Processes

Enzymes are biocatalysts in many industries (e.g., food, chemical, and pharmaceutical). The enzyme-assisted extraction processing (EAEP) simultaneously extracts oil and protein from plants. EAEP has a low environmental impact due to avoiding organic solvents and can be labeled as an eco-friendly technique that produces valuable products in mild conditions without losing quality (50).

EAEP causes plant cell walls disruption by specific enzyme activity (e.g., proteases, cellulases, and pectinases). These enzymes enhance the extraction yield by decoupling proteins attached to the plant polysaccharide matrix (50). Proteases are the most used enzymes for EAEP and bring the highest protein recovery (9, 11).

Besides the high extraction yield, EAEP can also improve the functional properties of plant proteins, depending on extraction conditions, such as temperature, pH, ionic strength, and presence of salts (93). High-quality co-products (e.g., polyphenols and polysaccharides) from plants can also be obtained with good yields and preserved intrinsic qualities using EAEP (93). Table 2 shows examples of EAEP applied to plant proteins.

In addition to enzyme-assisted extraction, the enzymatic hydrolysis (EH) technique may generate protein hydrolysates, such as di- and tri-peptides (9), and bioactive peptides from plant proteins, which contribute to the production of bio-functional foods (94, 95). EH is better than conventional chemical hydrolysis (e.g., acid or alkali) due to high product quality, reduced processing time, and mild conditions (17).

The endopeptidases (e.g., Alcalase^®^) and the exopeptidases (e.g., Flavourzyme^®^) used in the EAEP process break down some peptide bonds (17, 96). The resulting carboxyl groups and free amino acids increase the isolated proteins' nutritional value, digestibility, and functional properties (9, 49, 97). EH decreases molecular weight, increases hydrophilicity, and changes protein conformation (17, 97).

Multi-enzyme cocktails may be a strategy to improve hydrolytic reactions (96). The enhanced functionality in protein hydrolysates depends on the extent of hydrolysis and process conditions (e.g., pH, temperature, and enzyme/substrate ratio) (17). The degree of hydrolysis (DH) is the percentage of peptide bonds cleaved per gram of protein over the total number of peptide bonds (98). DH is crucial for obtaining desirable results and avoiding excessive protein hydrolysis that can impair functionalities and sensory attributes (38, 98).

EH can result in hypoallergenic foods due to peptides' production that does not trigger IgE antibody binding activity, lowering protein allergenicity (99). High-quality protein hydrolysates have applications in food formulations destined for individuals with special diets (e.g., infants, elderly, allergic individuals, or medical nutrition) or those seeking a higher protein intake (e.g., athletes) (17, 49). However, there are some challenges regarding protein hydrolysate applications. The significant drawbacks of protein hydrolysates are their palatability and the bitter taste due to the release of bitter hydrophobic peptides (8). Table 2 shows examples of applications of enzymatic hydrolysis to plant proteins.

### Other Emerging Technologies

Other processes commented in the literature allow the extraction and conformation changes, leading to high yield and improved protein quality. Examples are gamma irradiation (100), direct steam injection processing (DSI) (101), and refractance-window (RW) and cast-tape drying (CTD) (35).

DSI may lead to protein denaturation due to product exposure to high temperatures for short periods. DSI changes proteins' 3D conformation, like disulfide bonds and free S-H, and the protein surface becomes more hydrophilic, enhancing the functional properties (e.g., solubility) without affecting the essential amino acid composition (101).

CTD and RW are emerging techniques used for dehydrating liquid and semi-liquid foods (102, 103). The heat transfer occurs fastly to the product bottom by conduction (104). The processing time is relatively short, leading to minor changes in product nutritive value, conserving, or improving the protein functional properties (35).

Gamma irradiation (GI) may improve protein extraction and cause conformational changes (secondary structure rearrangement), crosslinking, and break covalent bonds. The protein structure and concentration and the presence of oxygen are the main factors affecting this process. However, GI may promote amino acid oxidation (8, 100).

Table 2 shows DSI, RW, and GI applications to plant proteins. There are rare studies on extracting plant proteins and evaluating their protein digestibility and techno-functional properties.

### Combined Emerging Technologies

Some studies show the combined effect of some presented emerging technologies (e.g., US, HPP, MH, OH) with the enzymatic processes on enhancing protein recovery, extraction yield, and improving protein functional properties (10, 105). The improvement of protein extraction and functional properties results can be explained due to the synergistic effect of the mentioned technologies related to the increase of mass transfer and the influence of the conformational and protein structure changes, for example. Table 3 shows applications of combined processes on plant proteins. These studies showed combined techniques as more efficient in enhancing protein functional properties. Nevertheless, more research is required to validate their application to improve the nutritional quality of plant proteins.

**Table 3:** Examples of combined processes on plant proteins.

**Application**	**Technique**	**Objective of the study**	**Processing conditions**	**Results**	**Protein yield**	**References**
Rapeseed meal	MH + EH	Time reduction	• 500 W • 7 min • 46°C	Improved protein hydrolysis and shorten the time from 4 h to 7 min.	^*^	(150)
Kidney bean protein isolate	HPP + EH	Functional properties	• 300 MPa • 15 min • Alcalase^®^ 1%	DH 23.9% and higher foaming capacity (90.3%)	^*^	(10)
Soybean protein isolate	US + EH	Functional properties	• 200–600 W • 25 kHz • 15 min • pH 7 • 55°C • 0.05–0.5% enzyme (papain)	Improved protein solubility, emulsifying capability, DH and surface hydrophobicity	^*^	(38)
Soybean	OH + EAEP	Oil recovery	• 600–900 V • 70–90°C • 5–10 min	Enhancement of oil recovery	^*^	(74)
Grapeseed	US + EH	Functional properties	• 20–50 kHz • 20 min • 30°C • Alcalase^®^ 0.5–50 g	Improved protein solubility	^*^	(105)

* *Data not found in the respective study*.

*DH, Degree of hydrolysis; EAEP, Enzyme-assisted extraction processing; EH, Enzymatic hydrolysis; HPP, High pressure processing; MH, Microwave heating; OH, Ohmic heating; US, Ultrasound*.

## Perspectives and Concluding Remarks

The emerging technologies for food processing are alternatives to traditional thermal processing to enhance plant proteins' nutritional quality or techno-functional properties. Protein quality is the main concern when replacing traditional sources, particularly meat by plants. It was observed that the selected processing technologies could interact with the protein structure (from primary to quaternary) and inactivate/eliminate the antinutritional factors, which indicate changes toward higher protein digestibility. This effect was demonstrated for high-pressure, ultrasound, microwave, ohmic heating, gamma irradiation, and enzymatic processes. In particular, trypsin inhibitors were inactivated by ultrasound, microwave, and ohmic heating, while gamma irradiation was suitable to inactivate the phytic acid. Protein allergenicity is also a concern for adding alternative protein sources in a diet, and it was demonstrated that enzymatic processes, cold plasma, and high-pressure were also efficient in reducing the allergenicity of these alternative proteins.

High-pressure processing is the most extensively explored process and presented many good results for techno-functional properties (foaming, emulsifying, and water- and oil-holding capacities), reducing allergenicity and antinutritional factors, and, as a consequence, improving plant protein digestibility and food applications. Those techno-functionality improvements result from changing the protein structure (from secondary to quaternary). Most of the emerging technologies discussed here can promote similar changes to the protein molecules, and, in this way, they are potential methods to impact those properties positively. In some studies, these properties were affected by ultrasound, pulsed electric field, microwave, cold plasma, direct steam injection, refractance-window drying, and enzymatic processes. In addition to the mentioned benefits, both ultrasound and enzymatic processes are suitable for increasing protein extraction yield and bioactive compounds recovery.

The promising results found about these technologies can be explored to nutritionally enrich traditional plant origin sources, such as soybean, peas, beans, and chickpeas. This work claims the broad perspective of these emerging technologies for achieving the adequate protein quality of plant origin sources. However, there are scarce studies evaluating protein digestibility and amino acid composition of plant proteins when using these emerging technologies. There is no published data on ohmic heating and cold plasma impact on plant protein quality enhancement or functional properties. Furthermore, combining some of these technologies has been indicated as a suitable strategy for better results, and many further investigations can be carried out to find optimal conditions.

Finally, the industrial application feasibility needs additional development at scales more extensive than those implemented at the laboratory. An actual industrial processing method must consider economic, environmental, and food security issues, which will supply sufficient proteins to the growing global population. Besides, the technological selection must meet human nutritional and sensory requirements and the consumer's cultural aspects. A key question is whether plant proteins can be extracted efficiently and cost-effectively and preserve nutritional value for human consumption worldwide.

## Author Contributions

AS conceived and designed the work, performed the data search, figure and tables preparation, and wrote the draft. JL and YM revised the draft. BC conceived and designed the work, and revised the draft. All authors contributed to the article and approved the submitted version.

## Funding

The authors thank the financial support from Brazilian Agencies CNPq (National Council for Scientific and Technological Development) and CAPES (Coordination for the Improvement of Higher Education Personnel—Financial code 001 and CAPES-PRINT Project 88887.310373/2018–00).

## Conflict of Interest

The authors declare that the research was conducted in the absence of any commercial or financial relationships that could be construed as a potential conflict of interest.

## Publisher's Note

All claims expressed in this article are solely those of the authors and do not necessarily represent those of their affiliated organizations, or those of the publisher, the editors and the reviewers. Any product that may be evaluated in this article, or claim that may be made by its manufacturer, is not guaranteed or endorsed by the publisher.
